# Supplementary material for: A Critical View of the Application of the APEX Software (Aqueous Photochemistry of Environmentally-Occurring Xenobiotics) to Predict Photoreaction Kinetics in Surface Freshwaters
Source: Molecules. 2019 Dec 18;25(1):9. doi: 10.3390/molecules25010009 (PMC7017383; doi:10.3390/molecules25010009)
Supplement: Supplementary file 1 [file molecules-25-00009-s001.zip › 3.2.4_gcc-4.4.0.zip!/3.2.4_gcc-4.4.0/3.2.4_gcc-4.4.0/share/octave/packages/gsl-1.0.8/doc/RCS/gsl.pdf,v]

err contains an estimate of the absolute error in the value *y*.

This function is from the GNU Scientific Library, see `http://www.gnu.org/software/gsl/`

y = exprel (*x*) [Loadable Function]

[*y*, *err*] = exprel ([Loadable Function]

err contains an estimate of the absolute error in the value *y*.

This function is from the GNU Scientific Library, see http://www.gnu.org/software/gsl/ for documentation.

ØShi -*- texinfo -*-

y = Shi (*x*) [Loadable Function]

[*y*, *err*] = Shi (...) [Loadable Function]

These routines compute the integral Shi(x) = int˙0^x dt sinh(t)/t.

err contains an estimate of the absolute error in the value *y*.

This function is from the GNU Scientific Library, see http://www.gnu.org/software/gsl/

[Loadable Function]

y = psi (*x*) [Loadable Function]

[*y*, *err*([Loadable Function]

y = `transport_3` (*x*) [Loadable Function]

[*y*, *err*] = `transport_3` (...) [Loadable Function]

These routines compute the transport function J(3,x).

The transport functions J(n,x) are defined by the integral representations J(n,x) := int˙0^x dt t^n e^t /(e^t - 1)^2.

err contains an estimate of the absolute error in the value *y*.

This function is from the GNU Scientific Library, see http://www.gnu.org/software/gsl/ for documentation.

Øtransport˙4 -*- texinfo -*-

y = `transport_4` (*x*) [Loadable Function]

[*y*, *err*] = `transport_4` (...) [Loadable Function]

These routines compute the transport function J(4,x).

The transport functions J(n,x) are defined by the integral representations J(n,x) := int˙0^x dt t^n e^t /(e^t - 1)^2.

err contains an estimate of the absolute error in the value *y*.

This function is from the GNU Scientific Library, see http://www.gnu.org/software/gsl/ for documentation.

Øtransport˙5 -*- texinfo -*-

y = `transport_5` (*x*) [Loadable Function]

[*y*, *err*] = `transport_5` (...) [Loadable Function]

These routines comp(u.t.e.t)he transport function J(5nsport˙4

ØIncosh -*- texinfo -*-

Øbessel˙yl -*- texinfo -*-

y = `bessel_yl` (*n, x*) [Loadable Function]

[*y*, *err*] = `bessel_yl` (...) [Loadable Function]

These routines compute the irregular spherical Bessel function of order l, y˙l(x), for l >= 0.

err contains an estimate of the absolute error in the value *y*.

This function is from the GNU Scientific Library, see http://www.gnu.org/software/gsl/ for documentation.

[Loadable Function]

ØconicalP˙mhalf -*- texinfo -*-

`z = conicalP_mhalf (x, y)` [Loadable Function]

`[z, err] = conicalP_mhalf (...)` [Loadable Function]

These routines compute the regular Spherical Conical Function P^{-1/2}˙{-1/2 + i lambnct

2 = GSL˙PREC˙APPROX
Approximate values, a relative accuracy of approximately 5 * 10^-4.

err contains an estimate of the absolute error in the value *y*.

y = airy_Bi_scaled (*x*, *mode*) [Loadable Function]
[*y*, *err*] = airy_Bi_scaled (...) [Loadable Function]

These routines compute a scaled version of the Airy function S˙B(x) Bi(x). For x>0 the scaling factor S˙B(x) is exp(-(2/3) x^(3/2)), and is 1 for x<0.

The second argument *mode* must be an integer corresponding to

0 = GSL˙PREC˙DOUBLE
: Double-precision, a relative accuracy of approximately `2 * 10^-16`.

1 = GSL˙PREC˙SINGLE
: Single-precision, a relative accuracy of approximately `10^-7`.

2 = GSL˙PREC˙APPROX
: Approximate values, a relative accuracy of approximately `5 * 10^-4`.

err contains an estimate of the absolute error in the value *y*.

This function is from the GNU Scientific Library, see http://www.gnu.org/software/gsl/ for documentation.

Øairy˙Ai˙deriv -*- texinfo -*-

y = airy_Ai_deriv (*x*, *mode*) [Loadable Function]
[*y*, *err*] = airy_Ai_deriv (...) [Loadable Function]

These routines compute vhe fnction isiv u(e)-334(ahe)-A isivwieisiv(y)-33curto9(f)-1(p)-27(ond)1

[Loadable FuBction]
[Loadable Function]

2 = GSL˙PREC˙APPROX
Approximate values, a relative accuracy of approximately 5 * 10^-4.

err contains an estimate of the absolute error in the value *y*.

This function is from the GNU Scientific Library, see http://www.gnu.org/software/gsl/ for documentation.

Øairy˙Ai˙deriv˙scaled -*- texinfo -*-

y = airy_Ai_deriv_scaled (*x*, *mode*) [Loadable Function]
[*y*, *err*] = airy_Ai_deriv_scaled [Loadable Function]

Øbessel˙zero˙J1 -*- texinfo -*-

y = bessel_zero_J1 (*n*) [Loadable Function]
[*y*, *err*] = bessel_zero_J1 (. . .) [Loadable Function]

These routines compute the location of the s-th positive zero of the Bessel function J˙1(x).

err contains an estimate of the absolute error in the value *y*.

This function is from the GNU Scientific Library, see http://www.gnu.org/software/gsl/ for documentation.

Øpsi˙1˙int -*- texinfo -*-

y = psi_1_int (*n*) [Loadable Function]
[*y*, *err*] = psi_1_int (. . .) [Loadable Function]

These routines compute the Trigamma function psi(n) for positive integer n.

err contains an estimate of the absolute error in the value *y*.

This function is from the GNU Scientific Library, see http://www.gnu.org/software/gsl/ for documentation.

Øzeta˙int -*- texinfo -*-

y = zeta_int (*n*) [Loadable Function]
[*y*, *err*] = zeta_int (. . .) [Loadable Function]

These routines compute the Riemann zeta function zeta(n) for integer n, n e 1.

err contains an estimate of the absolute error in the value *y*.

This function is from the GNU Scientific Library, see http://www.gnu.org/software/gsl/ for documentation.

Øeta˙int -*- texinfo -*-

y = eta_int (*n*)333(ze)-1(ta()1(n))-333(for)-333(in)29(te)-1(ger)-33[illegible] [Loadable Function][illegible](texin-333(-)-1(*-)][illegible]

y = legendre_sphPlm (*n*, *m*, *x*) [Loadable Function]
[*y*, *err*] = legendre_sphPlm ([Loadable Function]

`y = dawson (x)` [Loadable Function]
`[y, err] = dawson (...)` [Loadable Function]

The Dawson integral is defined by exp(-x^2) int_0^x dt exp(t^2). A table of Dawson integral can be found in Abramowitz & Stegun, Table 7.5.

err contains an estimate of the absolute error in the value *y*.

y = debye_4 (*x*) [Loadable Function]
[*y*, *err*] = debye_4 (...) [Loadable Function]

The Debye functions are defined by the integral

D˙n(x) = n/x^n int˙0^x dt (t^n/(e^t - 1)).

For further information see Abramowitz & Stegun, Section 27.1.

err

Øerf˙Q -*- texinfo -*-

y = erf_Q (*x*) [Loadable Function]
[*y*, *err*] = erf_Q (...) [Loadable Function]

y = exprel_2 (*x*) [Loadable Function]

[*y*, *err*] = exprel_2 (...) [Loadable Function]

These routines compute the quantity 2(exp(x)-1-x)/x^

ØChi -*- texinfo -*-

y = Chi (*x*) [Loadable Function]

[*y*, *err*] = Chi (*...*) [Loadable Function]

These routines compute the integral

Chi(x) := Re[gamma˙E + log(x) + int˙0^x dt (cosh[t]-1)/t] ,

where gamma˙E is the Euler constant.

err contains an estimate of the absolute error in the value *y*.

Øbessel˙kl˙scaled -*- texinfo -*-

y [Loadable Function]

z = hzeta (*x*, *y*) [Loadable Function]
[*z*, *err*] = hzeta (...) [Loadable Function]

These routines compute the Hurwitz zeta function zeta(s,q) for s > 1, q > 0.

err contains an estimate of the absolute error in the value *z*.

This function is from the GNU Scientific Library, see http://www.gnu.org/software/gsl/ for documentation.

Øairy˙Ai -*- texinfo -*-

y = airy_Ai (*x*, *mode*) [Loadable Function]
[*y*, *err*] = airy_Ai (...) [Loadable Function]

These routines compute the Airy function Ai(x) with an accuracy specified by mode.

The second argument *mode* must be an integer corresponding to

y = airy_Ai_scaled (*x*, *mode*) [Loadable Function]

[*y*, *err*] = airy_Ai_scaled (...) [Loadable Function]

These routines compute a scaled version of the Airy function S˙A(x) Ai(x). For x>0 the scaling factor S˙A(x) is exp(+(2/3) x^(3/2)), and is 1 for x<0.

The second argument *mode* must be an integer corresponding tomo 0

2 = GSL˙PREC˙APPROX

Approximate values, a relative accuracy of approximately `5 * 10^-4`.

err contains an estimate of the absolute error in the value *y*.

This function is from the GNU Scientific Library, see http://www.gnu.org/software/gsl/ for documentation.

Øairy˙Bi˙deriv -*- texinfo -*-

y [illegible] [Loadable Function]

[*y*, [illegible] [Loadable Function]

Øairy˙zero˙Bi˙deriv -*- texinfo -*-

y = airy_zero_Bi_deriv (*n*) [Loadable Function]
[*y*, *err*] = airy_zero_Bi_deriv (...) [Loadable Function]

These routines compute the location of the s-th zero of the Airy function derivative Bi(x).

err contains an estimate of the absolute error in the value *y*.

This function is from the GNU Scientific Library, see http://www.gnu.org/software/gsl/
